# Negative correlation between altitudes and oxygen isotope ratios of seeds: exploring its applicability to assess vertical seed dispersal

**DOI:** 10.1002/ece3.2380

**Published:** 2016-09-04

**Authors:** Shoji Naoe, Ichiro Tayasu, Takashi Masaki, Shinsuke Koike

**Affiliations:** ^1^ Forestry and Forest Products Research Institute Matsunosato 1 Tsukuba Ibaraki 305–8687 Japan; ^2^ Research Institute for Humanity and Nature 457‐4 Motoyama, Kamigamo Kita‐ku Kyoto 603‐8047 Japan; ^3^ Center for Ecological Research Kyoto University Hirano 2‐509‐3 Otsu Shiga 520‐2113 Japan; ^4^ Tokyo University of Agriculture and Technology 3‐5‐8 Saiwai Fuchu Tokyo 183‐8509 Japan

**Keywords:** Altitude, fruiting phenology, global warming, long‐distance seed dispersal, oxygen stable isotope, plant distribution, vertical seed dispersal

## Abstract

Vertical seed dispersal, which plays a key role in plant escape and/or expansion under climate change, was recently evaluated for the first time using negative correlation between altitudes and oxygen isotope ratio of seeds. Although this method is innovative, its applicability to other plants is unknown. To explore the applicability of the method, we regressed altitudes on *δ* ^18^O of seeds of five woody species constituting three families in temperate forests in central Japan. Because climatic factors, including temperature and precipitation that influence *δ* ^18^O of plant materials, demonstrate intensive seasonal fluctuation in the temperate zone, we also evaluated the effect of fruiting season of each species on *δ* ^18^O of seeds using generalized linear mixed models (GLMM). Negative correlation between altitudes and *δ* ^18^O of seeds was found in four of five species tested. The slope of regression lines tended to be lower in late‐fruiting species. The GLMM analysis revealed that altitudes and date of fruiting peak negatively affected *δ* ^18^O of seeds. These results indicate that the estimation of vertical seed dispersal using *δ* ^18^O of seeds can be applicable for various species, not just confined to specific taxa, by identifying the altitudes of plants that produced seeds. The results also suggest that the regression line between altitudes and *δ* ^18^O of seeds is rather species specific and that vertical seed dispersal in late‐fruiting species is estimated at a low resolution due to their small regression slopes. A future study on the identification of environmental factors and plant traits that cause a difference in *δ* ^18^O of seeds, combined with an improvement of analysis, will lead to effective evaluation of vertical seed dispersal in various species and thereby promote our understanding about the mechanism and ecological functions of vertical seed dispersal.

## Introduction

Seed dispersal is one of the few methods for plant movement and thus has several essential ecological functions. Local seed dispersal strongly influences population dynamics and thereby community dynamics: It provides opportunity for avoiding disproportionate seed and seedling mortality near the parent, colonizing after disturbance and locating microhabitats suitable for establishment and growth (Howe and Smallwood 1982). Long‐distance seed dispersal is much less studied as compared to the local one, but it influences population dynamics, evolution of populations, metapopulation dynamics, biological invasions, and the dynamics and diversity of ecological communities (Cain et al. 2000). The movement of dispersed seeds is expressed in three dimensions: horizontal dispersal toward latitudinal and/or longitudinal directions (*x*‐ and *y*‐axes), and vertical dispersal (i.e., dispersal toward lower/higher altitudes, *z*‐axis).

Vertical seed dispersal is considered to have important ecological functions on a large geographical scale. It shapes vertical distributions of plant communities in the mountains and facilitates gene flow among populations at lower and higher altitudes. In particular, it plays a key role in plant escape and/or expansion under climate change (Corlett and Westcott 2013; Naoe et al. 2016). This role becomes more important during intensive climate change including present global warming (ref. Briceño et al. 2015). Under present global warming, plants must disperse seeds several hundred kilometers along the latitudinal gradient until the year 2100 to keep up with the rapid rate of global warming (IPCC 2013): On the other hand, plants need to disperse seeds only several hundred meters along the vertical gradient due to the drastic decrease in temperature with increasing altitude (i.e., 100 m upslope roughly corresponds to −0.65°C) (Barry and Chorley 2009; also see Körner and Spehn 2002). Considering that long‐distance seed dispersal occurs at a very low frequency (Nathan 2006), it is very likely that plants rely on vertical seed dispersal to escape global warming. The finding that anthropogenic habitat fragmentation, which prevents seed dispersal [e.g., dispersal by animals (Cordeiro and Howe 2003; McEuen 2004; Naoe et al. 2011) and wind (Damschen et al. 2014)], is less severe in mountainous areas (Corlett and Westcott 2013; Naoe et al. 2015) highlights the reliability of vertical dispersal. Actually, plant distributions are tracking global warming altitudinally rather than latitudinally, and the extent of tracking is considered to be large in plants with better dispersal traits, such as lighter seed in wind‐dispersed plants (Parolo and Rossi 2008; Corlett and Westcott 2013). However, vertical seed dispersal itself has not been evaluated until recently (Naoe et al. 2016).

Two primary obstacles have made the evaluation of vertical seed dispersal difficult. First, drastic changes in many environmental factors occur with altitude, such as the climate and biome (Körner 2003). This prevents models from predicting vertical seed dispersal distance, because several essential modeling assumptions are not true with the altitudinal gradient. For example, in seed dispersal by wind, wind direction that determines the direction of seed dispersal is not spatially constant (Cousens et al. 2008); in seed dispersal by animal, animal movements that determine seed dispersal distance are limited by the animal's altitudinal range. In fact, vertical seed dispersal has never been modeled even in seed dispersal by wind, which is physically determined and thus, the causal factors have already been explored in detail (Nathan and Katul 2005). Second, observing vertical seed dispersal inevitably leads to observing long‐distance seed dispersal. Slope distance, corresponding to a particular vertical distance, is much longer than the vertical distance itself – alpinists must walk several kilometers to ascend several hundred meters vertically. It is very difficult to quantitatively measure such long‐distance seed dispersal at a high resolution from a practical point of view (intensive sampling of dispersed seeds and identifying their mother plants in a landscape or regional scale are needed) (Cain et al. 2000). Thus, a new and highly cost‐efficient method must be developed to evaluate vertical seed dispersal.

**Figure 1:**
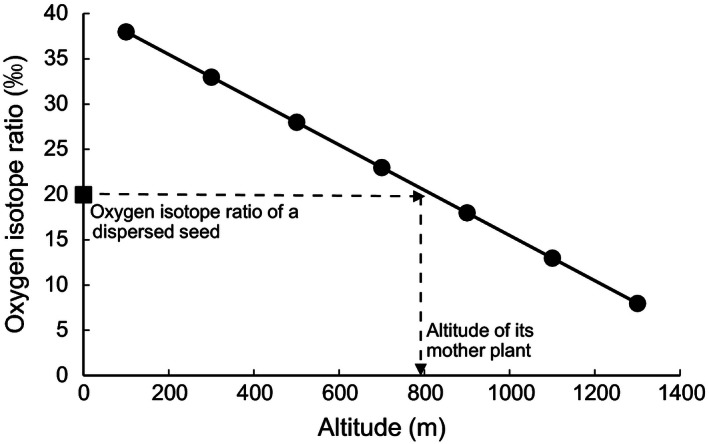
Hypothetical regression line between altitude and oxygen isotope ratio of seeds (solid line) and its use to estimate vertical seed dispersal. We can locate the altitude of a mother plant using the regression line and oxygen isotope ratio of a dispersed seed (square as an example) and thus can estimate vertical seed dispersal distance by subtracting the altitude of a mother plant from that of a dispersed seed.

Naoe et al. (2016) have recently evaluated vertical seed dispersal for the first time, using oxygen stable isotope ratios (^18^O/^16^O, here after *δ* ^18^O) of seeds. *δ* ^18^O of plants is originally based on *δ* ^18^O of precipitation, which decreases with altitude largely because of temperature‐driven distillation of the heavy isotope ^18^O from air masses as they move over orographic barriers (Bowen and Wilkinson 2002; Barbour 2007). Although plant internal factors, including evaporative enrichment of leaf water, isotopic exchange between water and organic molecules, also affect *δ* ^18^O of plants (Barbour 2007; Sternberg 2009), a decrease in the oxygen isotope ratios with altitude has been reported in plant materials such as leaves and tree rings (e.g., Burk and Stuiver 1981; Terwilliger et al. 2002). Naoe et al. (2016) found a negative correlation between altitudes and *δ* ^18^O of wild cherry seeds and then determined the altitude of the mother plant of a dispersed seed using *δ* ^18^O of the dispersed seed. As a result, they determined vertical seed dispersal distance, that is, the vertical distance between a dispersed seed and its mother plant (see Fig. 1 for the study concept). Their method is innovative in terms of simplicity and very low cost of evaluating vertical seed dispersal: It only needs the negative correlation between altitudes and *δ* ^18^O of nondispersed reference seeds, and dispersed seeds for estimating vertical seed dispersal. However, negative correlation between altitudes and *δ* ^18^O of seeds has been reported only in wild cherry and cultivated coffee (Rodrigues et al. 2011; Naoe et al. 2016), and thus, its applicability to other plants is unclear.

To explore the applicability of the method, we tested the relationship between altitudes and *δ* ^18^O of seeds of five woody species constituting of three families in temperate forests. Because climatic factors (e.g., temperature and precipitation), which affect *δ* ^18^O of plant materials, show intensive seasonal fluctuation in the temperate zone (Barry and Chorley 2009), we also evaluated the effect of the fruiting season of each species on *δ* ^18^O of seeds.

## Material and Methods

**Figure 2:**
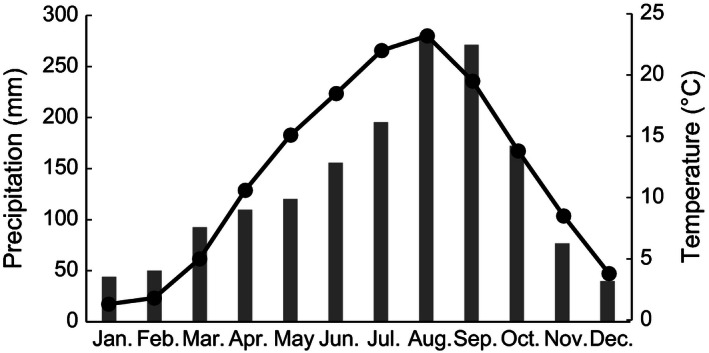
Monthly mean precipitation and temperature from 1981 to 2010 at a nearest meteorological station located 2 km from the study site, 530 m a.s.l. (Japan Meteorological Agency 2016). Bars and solid line indicate precipitation and temperature, respectively.

This study was conducted at Okutama in the Kanto region, approximately 100 km west of Tokyo, Japan. The climate in the study area is the Japanese Pacific Ocean type, with heavy rainfall in summer and little snow in winter. The mean annual precipitation during 1981–2010 at 530 m a.s.l. was 1624 mm, and the mean annual temperature was 11.9°C (range: 1.3°C in January to 23.2°C in August) (Fig. 2) (Japan Meteorological Agency 2016). The study area is mountainous and is covered mostly with forest vegetation. Natural forests and conifer plantations (*Cryptomeria japonica* or *Chamaecyparis obtusa*) cover 41.3 and 50.3% of the area, respectively. The natural forests are dominated by *Castanea crenata* and *Quercus serrata* in the lower mountain zone (400–500 m a.s.l.); by *Quercus crispula*,* C. crenata*, and *Fagus crenata* in the middle zone (500–1500 m a.s.l.); and by *Abies homolepis* and *Tsuga diversifolia* in the upper zone (1500–1800 m a.s.l.) (Koike et al. 2008).

We targeted five fleshy fruited deciduous woody species of thee family, *Prunus jamasakura*,* P. verecunda*,* P. grayana*,* Swida controversa*, and *Actinidia arguta*, in the descending order of flowering and fruiting seasons (Table 1). These are widespread and rather abundant mountain species and thus are suitable for obtaining a wide vertical sampling range. We collected seeds directly from fruiting trees in various altitudes in their fruiting season during 2012–2014 in the radius of 10 km. Some of the samples of *P. verecunda* in 2012–2014 had also been used previously (Naoe et al. 2016). We analyzed three seeds per fruiting tree. We used the most external dead and hard tissue of a diaspore as the analysis subject, considering the possibility that *δ* ^18^O of whole seed demonstrates diurnal variation likely because of live tissues in the seeds (ref. Cernusak et al. 2002). Consequently, we analyzed the endocarp of all species except *A. arguta,* for which we analyzed its seed coat. In the study of *δ* ^18^O of plant materials, it is now rather common to extract *α*‐cellulose for purification and analyze it (ref. Barbour 2007). However, we used raw materials for efficiency, referring to Barbour et al. (2001), who indicated that when a wide range in temperature and *δ* ^18^O of precipitation exists (expected situation in our study), much information may be gained from *δ* ^18^O of raw materials as compared to that from *α*‐cellulose. By skipping this process, we could analyze *A. arguta* seeds, which otherwise were too small to analyze. The endocarp or seed coat was extracted from the seed and grinded for subsequent oxygen isotope analysis (Iacumin et al. 2009). Each endocarp or seed coat was weighed (approximately 0.15 mg) into silver capsules (Säntis Analytical, Milan, Italy) and rolled into balls for continuous flow (CF) combustion and isotope ratio mass spectrometry (IRMS) analysis using a high‐temperature elemental analyzer (TC/EA, Thermo Fisher Scientific, Waltham, Massachusetts, USA) coupled online with a mass spectrometer (Delta V plus; Thermo Fisher Scientific) using a ConFlo IV interface (Thermo Fisher Scientific). The molecular water absorbed from the atmosphere was removed in advance by vacuum‐drying the samples for at least one night before measurements to avoid water contamination in the samples. The oxygen isotope analyses were performed by measuring the CO obtained by high‐temperature carbothermic reduction of the endocarp (1375°C) in the presence of excess of carbon by means of a TC/EA. Before entering into the mass spectrometer, the helium stream containing CO was separated from H_2_ and N_2_ by a 1.4‐m molecular sieve chromatographic column held at 90°C. The sample gases were calibrated by measuring the reference substances (IAEA‐601 and IAEA‐602 benzoic acid; 23.14‰ and 71.28‰, respectively) of known isotope composition (Brand et al. 2009). The isotopic composition of a sample is conventionally expressed as “*δ*” value in per mill by comparison with international primary reference material (Vienna Standard Mean Ocean Water, V‐SMOW) as follows:

$$\delta^{18}O=R_{\mathrm{sample}}/R_{\mathrm{reference}}-1$$

where *R* denotes the ratio of numbers (*N*) of each isotope

$$R=N\left({}^{18}O\right)/N\left({}^{16}O\right)$$

**Table 1:** Family, fruiting season, life form, analyzed tissue in this study, sampling year, and altitude of target woody species

Species	Family	Flowering season	Fruiting peak^a^	Life form	Analyzed tissue	Sampling year	Sampling altitude
*Prunus jamasakura*	Rosaceae	May	Early July (191)	Canopy tree	Endocarp	2012	280, 570, 1100 m
*Prunus verecunda*	Rosaceae	May	Mid July (198)	Canopy tree	Endocarp	2012	550, 800, 1100, 1290 m
						2013	550, 800, 1000, 1100, 1180, 1290 m
						2014	550, 1000, 1110, 1180 m
*Prunus grayana*	Rosaceae	May	Early Sep. (251)	Subcanopy tree	Endocarp	2014	260, 590, 940, 1110, 1180, 1290 m
*Swida controversa*	Swidaceae	May	Late Sep. (265)	Canopy tree	Endocarp	2012	350, 680, 900 m
*Actinidia arguta*	Actinidiaceae	June	Late Oct. (295)	Woody vine	Seed coat	2013	600, 950, 1100, 1200, 1280 m

a Data from Masaki et al. (2012) and Naoe personal observation at ca. 650 m a.l.s. in a deciduous forest in the Kanto region. The number in the parenthesis indicates elapsed days from January 1.

The standard deviation of the replicates was approximately 0.2‰ (1*σ*) for measurements.

To estimate the calibration line for each species, we regressed *δ* ^18^O of seeds produced in the same year and altitudes (Table 1), considering the possibility of *δ* ^18^O variation among years (Naoe et al. 2016). To evaluate the effect of the fruiting season on *δ* ^18^O of seeds, we made generalized linear mixed model (GLMM), for which we used *δ* ^18^O of each seed as a response variable, altitude and fruiting peak (elapsed days from January 1st, maximum of 365) as explanatory variables, and species identity and year as random factors. We used the day of fruiting peak, assuming that the timing of fruit maturation is the same with that of seed maturation. Because we had no information regarding the exact day of fruiting peak at the study site and because fruiting peak of the same species was different among altitudes, we used the fruiting peak measured in a temperate forest in the same Kanto region at 650 m a.s.l. (Masaki et al. 2012). We normalized the explanatory variables for analysis. Correlation between both explanatory variables was <0.7, which indicates that multicollinearity is not severe (Dormann et al. 2013). In the analysis, we used Gaussian distribution for error and the function lemr in the lme4 package (Bates et al. 2014) in R (R Core Team 2014).

## Results

**Figure 3:**
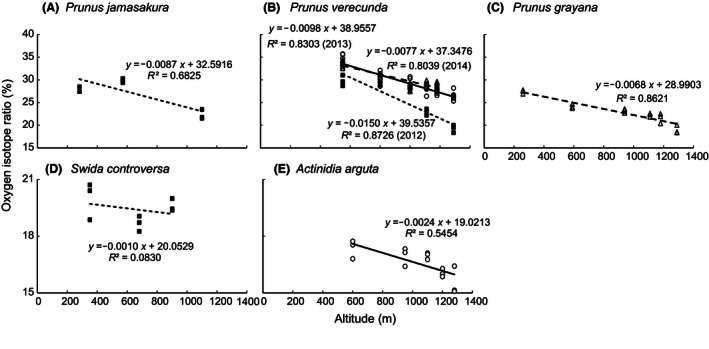
Relationship between altitude and oxygen isotope ratio of seeds of (A) *Prunus jamasakura*, (B) *Prunus verecunda*, (C) *Prunus grayana*, (D) *Swida controversa*, and (E) *Actinidia arguta* in ascending order of the date of fruiting peak. Squares, circles, and triangles indicate seeds sampled in 2012, 2013, and 2014, respectively. Note that regression lines were estimated for each year (2012, dotted line; 2013, solid line; 2014, dashed line) and that y‐axes are different in the upper (A–C) and lower graphs (D, E).

A negative correlation between altitudes and *δ* ^18^O of seeds was found in four of the five species tested (Fig. 3). The slope of regression lines tended to be lower in late‐fruiting species. The GLMM analysis revealed that altitudes and date of fruiting peak negatively affected *δ* ^18^O of seeds (Table 2).

**Table 2:** Result of the GLMM, showing coefficient, standard error (SE), and *t*‐ and *P*‐values for each explanatory variable

Explanatory variables	Coefficient	SE	*t*	*P*
Altitude	−2.283	0.126	−18.190	<0.001
Date of fruiting peak	−4.212	0.279	−15.100	<0.001

## Discussion

Significant regression lines between altitudes and *δ* ^18^O of seeds among multiple taxa indicate that the estimation of vertical seed dispersal using *δ* ^18^O of seed can be applicable for various species, not just confined to specific taxa. Although we targeted only temperate woody species, our method will not be confined to temperate plants because this is based on the negative correlation between altitudes and *δ* ^18^O of precipitation that is observed worldwide (Bowen and Wilkinson 2002). In fact, in the study on the identification of the geographical origin of food stuffs, a negative correlation between altitudes and *δ* ^18^O of cultivated coffee bean was reported in tropical Hawaii (Rodrigues et al. 2011). The fact that many plant species distribute in wide vertical range – over 500 m range is quite common, and over 1500 m range is not rare (e.g., in temperate and subarctic zone, Horikawa 1972; Flora of North America Editorial Committee 1997; in tropical zone, Soepadmo et al. 1996) – is also favorable for making a regression line between altitude and *δ* ^18^O of seeds. There are not negligible exceptional species which have short vertical ranges because of preferring specific habitat such as the coast. However, to begin with, these species have almost no chance to establish their populations at higher altitudes where their suitable habitats are probably not available. By applying our method for representative plants in each climatic zone, it may be possible to promote elucidating the process that determines the distribution of each species and plant community, and to upgrade the prediction of their future distributions under global warming. Significant regression lines between altitudes and *δ* ^18^O of seeds were not observed in *Swida controversa*. There are two potential reasons for this failure. The first is poor sampling design for *S. controversa*: Its sampling vertical range and locations were smallest among the five species (i.e., 550 m range and three locations). This might lower the statistical power. The second is species‐specific traits such as the fruiting season. The regression slopes of *S. controversa* may become very small due to intense variation in climate during its seed maturation (see the detailed discussion below). In such cases, a combination of other stable isotopes and elements for locating the mother plants (Rodrigues et al. 2011; Webb‐Robertson et al. 2012) may be useful for evaluating vertical seed dispersal.

While we could gain effective regression lines from four of five species, there were remarkable differences among the species in *δ* ^18^O of seeds and in the regression slope: *δ* ^18^O of seeds and regression slope were smaller in late‐fruiting species. This suggests that it is not easy to apply regression lines of other species for estimating vertical seed dispersal, even if they belong to the same genus. As for the difference between *δ* ^18^O of seeds among species, the timing of seed maturation, which is closely connected to climatic factors, would be the reason. Low temperature, high precipitation, and relative humidity are known to lower *δ* ^18^O of plant tissues (Barbour 2007). At the study site, late‐fruiting species experienced higher temperature and precipitation during their seed maturation as compared with early‐fruiting species (i.e., July to September, Fig. 2). Thus, although we lack information regarding relative humidity, seasonal variation in precipitation might cause the difference between *δ* ^18^O of seeds among species. As for the difference between the regression slopes among species, the length of seed maturation may be the reason. In the present study, the length of seed maturation of late‐fruiting species is longer than that of early‐fruiting species because all species flower in spring (Table 1). Therefore, late‐fruiting species experienced drastic climate variation during their seed maturation (Fig. 2). For instance, temperature change from July to August in a nearest meteorological station is 4.7°C which corresponds to 723 m in altitudinal distance following the lapse late (i.e., 100 m upslope roughly corresponds to −0.65°C) (Barry and Chorley 2009). The slopes of late‐fruiting species may become smaller because climate variation during their seed maturation surpasses the climate variation with altitude. This suggests the low resolution of vertical seed dispersal in late‐fruiting species due to small regression slopes. But it is notable that we used the date of fruit maturation instead of seed maturation in the analysis. There were mature seeds in mature fruits in all species, but the date of seed maturation might be faster than that of fruit maturation. It will be desirable to clarify the exact timing of seed maturation for a finer explanation for *δ* ^18^O of seeds.

While our results indicate the potential applicability for various plants and reveal the effect of fruiting season on the altitude–*δ* ^18^O relationship of seeds, it is notable that our results have several caveats stemming from too little information about *δ* ^18^O of seeds. First, the mechanism of how altitude affects *δ* ^18^O of seeds remains ambiguous. In a future study, we need to test what climatic and other environmental factors affect *δ* ^18^O of seeds by investigating the relationships between these factors and *δ* ^18^O of seeds. To this end, seed sampling across a long chronosequence or various locations that include multidimensional environmental gradient will be effective. Second, it may be problematic that we focused on only the fruiting season, which is closely linked to seasonal variation in climate, as the causal factor for the difference in *δ* ^18^O of seeds among species. Other plant traits may also cause differences among species. For example, species‐specific root structure and habitat may affect *δ* ^18^O of seeds through the differences in *δ* ^18^O of source water that the plants take up (ref. Flanagan et al. 1992; Roden and Ehleringer 2000; Yamanaka et al. 2006). In addition, species‐specific differences in seed chemical composition (ref. Gray and Thompson 1977) and in physical distance from vein to apoplast and tortuosity of the diffusive pathway that drive evaporative enrichment of *δ* ^18^O of leaf water (Barbour 2007; Sternberg 2009) are likely to affect *δ* ^18^O of seeds. Although we believe that the effect of the fruiting season on *δ* ^18^O of seeds is robust to some extent considering that the effect was detected among multiple taxa, we need to test whether this effect can be detected among taxa that vary greatly in other plant traits. Annual *δ* ^18^O of precipitation at study sites estimated from global database OIPC (Online Isotopes in Precipitation Calculator, Bowen and Revenaugh 2003) were ranging from −7.7‰ at 200 m a.s.l. to −9.9‰ at 1300 m a.s.l., decreasing −0.2‰ with altitude increasing 100 m. Their much lower values compared to that of seeds (range from 15.1 to 35.7‰), decreasing −1.3 to −0.2‰ with altitude increasing 100 m (Fig. 3), suggests strong but unidentified isotopic effects on *δ* ^18^O of seeds before seed maturation. The identification of environmental factors and plant traits that cause differences in *δ* ^18^O of seeds among altitudes and among species, combined with improvement of analysis, will lead to evaluation of vertical seed dispersal at a high resolution in various species and thereby promote our understanding about the mechanism and ecological functions of vertical seed dispersal.

## Conflict of Interest

None declared.
